# Economic Benefits of Care Coordination for Older Adults

**DOI:** 10.1093/geroni/igaf122.3507

**Published:** 2025-12-31

**Authors:** Erblin Shehu, Brian Kaskie

**Affiliations:** State University of New York at Cortland, United States; University of Iowa, Iowa City, Iowa, United States

## Abstract

In response to rising costs associated with providing healthcare services to Americans over 65 years old, policymakers have called for the expansion of care coordination programs to reduce total spending while improving patient outcomes. This study estimates the financial impact of the Iowa Return to Community (IRTC) initiative, a care coordination program designed to identify at-risk older adults and support their transitions from nursing homes and hospital settings to community living. We apply a Markov chain model with Monte Carlo simulations to capture all possible transitions among hospitals, nursing homes, and community settings. Using de-identified data from the State of Iowa (N = 523; 2020–2022) and a statistically matched control group from the National Health and Aging Trends Study (NHATS, 2020–2021), we compare IRTC outcomes with projections of what would occur without the program. Matching criteria include race, ethnicity, gender, income, living status, and 12 measures of instrumental (IADLs) and basic (ADLs) activities of daily living. Findings suggest IRTC participation is associated with reduced healthcare service utilization, resulting in per capita cost savings of $7,920.24 over five years. Subgroup analysis reveals that including informal care partners (ICPs) further increases these savings, as their involvement was linked to lower inpatient hospitalizations and deferred nursing home admissions. Findings call for IRTC expansion as a viable strategy to reduce aggregate healthcare spending while supporting aging in place.

